# Maximum *a posteriori* Threshold Genomic Prediction Model for Ordinal Traits

**DOI:** 10.1534/g3.120.401733

**Published:** 2020-09-15

**Authors:** Abelardo Montesinos-López, Humberto Gutierrez-Pulido, Osval Antonio Montesinos-López, José Crossa

**Affiliations:** * Departamento de Matemáticas, Centro Universitario de Ciencias Exactas e Ingenierías (CUCEI), Universidad de Guadalajara, 44430, Jalisco, México; † Facultad de Telemática, Universidad de Colima, 28040, México; ‡ Biometrics and Statistics Unit, International Maize and Wheat Improvement Center (CIMMYT), Carretera Km 45, Mexico-Veracruz, CP 52640, Texcoco, México; § Colegio de Postgraduados, CP 56230, Montecillos, Edo. de México

**Keywords:** maximum *a posteriori* estimation, EM algorithm, Bayesian Threshold Genomic Prediction model, support vector machine, multinomial Ridge regression, genomic selection, Genomic Prediction, GenPred, Shared data resources

## Abstract

Due to the ever-increasing data collected in genomic breeding programs, there is a need for genomic prediction models that can deal better with big data. For this reason, here we propose a Maximum *a posteriori* Threshold Genomic Prediction (MAPT) model for ordinal traits that is more efficient than the conventional Bayesian Threshold Genomic Prediction model for ordinal traits. The MAPT performs the predictions of the Threshold Genomic Prediction model by using the maximum *a posteriori* estimation of the parameters, that is, the values of the parameters that maximize the joint posterior density. We compared the prediction performance of the proposed MAPT to the conventional Bayesian Threshold Genomic Prediction model, the multinomial Ridge regression and support vector machine on 8 real data sets. We found that the proposed MAPT was competitive with regard to the multinomial and support vector machine models in terms of prediction performance, and slightly better than the conventional Bayesian Threshold Genomic Prediction model. With regard to the implementation time, we found that in general the MAPT and the support vector machine were the best, while the slowest was the multinomial Ridge regression model. However, it is important to point out that the successful implementation of the proposed MAPT model depends on the informative priors used to avoid underestimation of variance components.

In plant breeding it is very common to measure ordinal traits like gray leaf spot (GLS) resistance (0 = no infection, 1 = low, 2 = medium, 3 = high, 4 = total infection level) (Montesinos-López *et al.*, 2015), rice sheath blight resistance measured on a 0-9 scale, where 0 indicates no disease and 9 indicates dead or collapsed plants (Zou *et al.*, 2000), cucumber mosaic virus (CMV) resistance (1 = no symptoms on the third and fourth axillary shoots, 2 = systemic necrosis on the shoots and/or mosaic on the leaves of the third axillary shoot [corresponding to the inoculated leaf], 3 = systemic necrosis on the shoots and/or mosaic on the leaves of both axillary shoots) (Caranta *et al.*, 2002), etc. For this reason, appropriate genomic selection (GS) methods for dealing with ordinal traits have been developed, for example, the Bayesian Threshold Genomic Best Linear Unbiased Predictor (TGBLUP) proposed by Montesinos-López *et al.* (2015). However, the TGBLUP model requires considerable computational resources since it was built under a Bayesian framework and involves the sampling process of high-dimensional unknown parameters iteratively. The TGBLUP model is a Bayesian version of classical probit models which were first introduced by Bliss (1934a, b) and Gaddum (1933) for binary data.

The TGBLUP is very competitive in terms of prediction performance, as was shown by Montesinos-López *et al.* (2019), who compared this method to deep learning (DL) and support vector machine (SVM). However, due to the fact that the TGBLUP model was built under a Bayesian framework (that uses Gibbs sampling), it requires a lot of computational resources because convergence requires considerable time in the context of large data sets. For this reason, methods for ordinal data that are more cost effective than Markov Chain Monte Carlo (MCMC) sampling are lacking. One approach to partially solve this problem of MCMC, is to base the prediction in terms of different point estimates of the parameters, for example, by using the maximum *a posteriori* (MAP) estimate of the parameters, which maximizes their joint posterior distribution. This is different from the MCMC framework where the full posterior probability distribution is explored and then summarized (mean, median, quantiles, etc.) to draw inferences and make predictions. The MAP also uses the full posterior distribution, f(θ|y)∝f(y|θ)f(θ), that contains all the knowledge about the unknown quantity θ to find point or interval estimates of θ, but instead the MAP solves an optimization problem to estimate a central tendency (point estimate) of the posterior probability; the θ values that maximize the full posterior distribution, f(y|θ)f(θ), are called the MAP estimates. For the latter, the MAP estimator is interpreted as an analog of maximum likelihood for Bayesian estimation, since instead of maximizing the likelihood, it maximizes an augmented likelihood, that is, the posterior distribution.

For this reason, the MAP is an alternative probability framework for Bayesian methods under the MCMC framework. It selects the most likely hypothesis given the data and a prior distribution of the parameters, is often more tractable than full Bayesian learning, requires less computing time than MCMC methods, and can be implemented for large data sets more efficiently. Also, the larger the data set, the better its performance (Brownlee 2019). However, the posterior means (pure Bayesian) are always preferred over the MAP estimates under a theoretical point of view. But when the posterior is not in closed form or is difficult to sample, MAP estimators can be calculated much faster in several orders of magnitude than posterior means. It is also important to point out that if the posterior is approximately symmetric (more common with larger data sets), MAP estimates are closer to posterior means and can be a good point estimate (Gelman *et al.*, 2014). So, the attractiveness of the MAP is actually that it can be a very cheap approximation of the posterior mean. One of the drawbacks of the MAP method is that it does not allow estimating uncertainty in the parameters (SE, variance, etc.), which is a big deal in association studies, but not a big problem in the prediction paradigm, since for the evaluation of prediction performance, we can use cross-validation and the bootstrap method to estimate the uncertainty of the parameter estimates.

Applications of the MAP in statistical science for association and prediction studies are many, for example, for the estimation of item parameters and latent abilities in item response theory (Rigdon and Tsutakawa 1983), for estimation in a multivariate normal regression model with incomplete data (Meng and Rubin 1993), for parameter estimation in a gamma model with incomplete data (Meng and Rubin 1993), for parameter estimation in mixed models in the presence of missing data in quantitative genetics (Lindstrom and Bates 1988; Van Dyk 2000), for parameter estimation in probit models (Ruud 1991), for the estimation of the polychoric correlation when two ordinal items were measured (Chen and Choi 2009) and for image reconstruction (Dong 2007; Hebert and Leahy 1989).

In genomic selection, MAP strategy has been applied for continuous traits and ordinal traits. For example, Yi and Banerjee (2009) considered MAP estimation appropriate for generalized linear models, but not appropriate for continuous traits. Shepherd *et al.* (2010) developed a MAP estimation for the BayesB model, which is a different formulation than the BayesB estimation done by Hayashi and Iwata (2010), who also considered a MAP estimation of the BayesA model. Kärkkäinen and Sillanpää (2013) developed a MAP estimation for the ordinal model with a Laplace prior distribution of the marker effects.

Parameter estimation using the MAP approach is straightforward when the f(θ|y)∝f(y|θ)f(θ) has a closed form since an analytical solution can be obtained using standard calculus techniques. However, this case is rare even when we have all the full conditionals for each component of θ. For this reason, most of the time the following are used for MAP estimation: (a) numerical methods (Newton’s methods, conjugate gradient descendent, etc.) that need first or second order derivatives, (b) the Expected Maximization (EM) algorithm that does not require derivatives of the posterior density, and (c) the Monte Carlo method using simulated annealing.

Of the three options, the EM algorithm can be a good alternative in some problems, since it does not require derivatives of the full posterior distribution and iteratively allows finding parameter estimates in the presence of missing data and unobserved (hidden) random variables in the models, and when the random variables belong to the exponential family, its performance is guaranteed.

The EM algorithm maximizes a lower bound of the likelihood function or augmented likelihood, better known as the Q-function, iteratively. Two steps are performed at each iteration: the Expectation (E) and Maximization (M) steps. The Q-function that consists of integrating out the missing values, allows obtaining the expected value of the complete data log likelihood function (observed + missed), while the M step consists of maximizing the Q-function over the unknown parameters. This iterative process is repeated until the convergence criterion is satisfied. Due to the fact that the maximization step most of the time is computationally simple because it only involves complete data, and that its convergence is stable, the EM algorithm enjoys great popularity. The EM algorithm is safe since it guarantees an increasing likelihood sequence and safe monotonic convergence (McLachlan and Krishnan 1997; Dempster *et al.*, 1977; Borman 2004). However, although the EM algorithm converges toward a stationary point of the marginal posterior density, (a) it depends on initialization, (b) it is a deterministic algorithm since it does not allow automatically estimating a variance-covariance matrix of parameter estimates (the uncertainty), and (c) it is limited to models where it is possible to conveniently perform the expectation and maximization steps.

However, to broaden the applicability of the EM algorithm to circumstances where the M-step is more complicated, Meng and Rubin (1993) extended the conventional EM to complicated M-steps by replacing the M-step of the EM algorithm with a sequence of conditional maximization (CM) steps in which each component parameter is maximized individually, conditionally on the other parameters remaining fixed. Meng and Rubin (1993) called this extension the Expectation conditional maximization (ECM) algorithm.

The ECM algorithm is attractive as a tool for predicting ordinal data in the context of genomic selection since the data sets collected for plant breeding continue growing, and also because there is empirical evidence that the difference in speed between Bayesian models under MCMC and a MAP estimation algorithm is far from trivial. The run time of an MCMC algorithm is typically hours at the lowest, while EM algorithms perform the analyses in significantly less time (Kärkkäinen and Sillanpää 2013). For this reason, in this paper we propose an expected conditional maximization *a posteriori* threshold (MAPT) model for parameter estimation in the Threshold Genomic prediction model.

Our proposed method is different from the GEM algorithm that Kärkkäinen and Sillanpää (2013) used to analyze ordinal genomic data, since their algorithm works by updating each parameter with the expected values of the corresponding fully conditional posterior distribution, while we use the conditional mode of each parameter and also a different latent variable.

## Material and Methods

### Statistical models

#### Bayesian threshold genomic best linear unbiased prediction (TGBLUP):

The ordinal probit model assumes that conditioned to $x_{i}$ (covariates of dimension p), $Y_{i}$ is a random variable that takes values ​​1, ..., C, with the following probabilities:

$$\begin{array}{l} P\left(Y_{i}=\text{c}\right)=P\left(\gamma_{c-1}\leq l_{i}\leq \gamma_{c}\right)\\ =\text{Φ}\left(\gamma_{c}+x_{i}^{T}\beta \right)-\text{Φ}\left(\gamma_{c-1}+x_{i}^{T}\beta \right),\;c=1,\;\ldots ,\;C \end{array}$$ (1)

where $\beta ={\left(\beta_{1},\ldots ,\beta_{p}\right)}^{T}$ are beta coefficient effects associated with the p explanatory variables, and $-\infty =\gamma_{0}<\gamma_{1}<\;\ldots <\gamma_{C\;}=\infty$ are threshold parameters. A Bayesian formulation of this model assumes the following independent priors for the parameters: a flat prior distribution for $\gamma =\left(\gamma_{1},\ldots ,\gamma_{C-1}\right)$ (f(γ)∝1), a normal distribution for beta coefficients, $\beta_{j}|\sigma_{\beta }^{2}∼N\left(0,\sigma_{\beta }^{2}\right)$, j=1,…, p, and a scale inverse chi-squared distribution for $\sigma_{\beta }^{2}$, $\sigma_{\beta }^{2}∼\chi_{v_{\beta },S_{\beta }}^{-2}$. The same prior variance is assigned to all independent covariates, so the shrinkage is homogeneous.

This threshold model assumes that the process that gives rise to the observed categories is an underlying or latent continuous normal random variable $l_{i}=-x_{i}^{T}\beta +\epsilon_{i}$ where $\epsilon_{i}$ is a normal random variable with mean 0 and variance 1, and the values of $l_{i}$ are called “liabilities” (Gianola, 1982, and Sorensen *et al.*, 1995). The ordinal categorical phenotypes in model (1) are generated from the underlying phenotypic values, $l_{i}$, as follows: $y_{i}=1$ if $-\infty <l_{i}<\gamma_{1},\;y_{i}=2$ if $\gamma_{1}<l_{i}<\gamma_{2},$…., and $y_{i}=C$ if $\gamma_{C-1}<l_{i}<\infty$. The TGBLUP model can be implemented in the BGLR package of Pérez-Rodríguez and de los Campos (2014) in the R statistical software (R Core Team 2020).

#### Multinomial ridge regression:

The multinomial Ridge regression model, with C levels for the response variable, c=1,2,..,C, assumes the following relation with a regressor variable x:

$$P\left(Y=c\text{|}x\right)=\frac{\text{exp}(\beta_{0c}+\beta_{c}^{T}x)}{\sum_{k=1}^{C}\text{exp}(\beta_{0k}+\beta_{k}^{T}x)}$$ (2)

Let Y be the n×C indicator response matrix, with elements $Y_{ic}=I(Y_{i}=c)$. Then the Ridge penalized log-likelihood function becomes:

$$\begin{array}{l} l_{p}\left(\beta_{0},\beta \right)=\frac{1}{n}\overset{n}{\underset{i=1}{\sum }}\left\{\overset{C}{\underset{c=1}{\sum }}y_{ic}\left(\beta_{0c}+x_{i}^{T}\beta_{c}\right)-\log\left[\overset{C}{\underset{c=1}{\sum }}\exp\left(\beta_{0c}+x_{i}^{T}\beta_{c}\right)\right]\right\}\\ \;\;\;\;\;\;\;\;\;\;\;\;\;\;\;\;\;\;\;\;-\frac{1}{2}\lambda {\left\|\beta \right\|}^{2} \end{array}$$ (3)

where $\beta_{0}=\left(\beta_{01},\ldots ,\beta_{0C}\right)$, β is a p×C matrix of coefficients with column c equal to $\beta_{c}$, regression coefficients are related to outcome category c, c=1, 2, ..., C, λ≥0 is a regularization parameter that determines how much the beta coefficients are shrunk toward zero. The optimization of this loss function (3) can be done using the R package glmnent (Lasso and Elastic-Net Regularized Generalized Linear Models) (Friedman *et al.*, 2010). To select the tuning hyperparameter (λ), this function performs a 10-fold cross-validation with the training set. This default strategy will be used in the applications.

#### Support vector machine:

Support Vector Machine (SVM) is a popular and efficient machine learning algorithm proposed by Vapnik (1995) for binary classification problems. Its versatility and the fact that it performs well in the presence of a large number of predictors, even with a small number of cases, makes SVM very appealing for solving a great variety of problems such as text categorization, image recognition, speech recognition, face detection, faulty card detection, credit rating analysis, junk mail classification, diabetes classification and cancer, to mention some of them (Byun and Lee 2002; Attewell *et al.*, 2015). SVM is the solution to the following optimization problem in its dual representation:

$$\underset{\alpha }{\underset{︸}{\text{Maximize}\;}}L\left(\alpha \right)=\overset{n}{\underset{i=1}{\sum }}\alpha_{i}-\frac{1}{2}\overset{n}{\underset{i=1}{\sum }}\overset{n}{\underset{j=1}{\sum }}\alpha_{i}\alpha_{j}y_{i}y_{j}K\left(x_{i},x_{j}\right)$$ (4)

$$\text{subject to}:\;\overset{n}{\underset{i=1}{\sum }}\alpha_{i}y_{i}=0\;\text{and}\;0\leq \overset{n}{\underset{i=1}{\sum }}\alpha_{i}\leq T$$ (5)

where T is a non-negative tuning parameter that determines the number and severity of violations to the margin (and to the hyperplane) that we will permit; it is seen as the total amount of errors that will be tolerated. Generally, this is chosen by cross-validation. $K\left(x_{i},x_{j}\right)$ is a kernel, which is a positive definite function that quantifies the similarity between two observations (James *et al.*, 2013).

Once found, the α value in the optimization problem in (4) and (5), $\hat{\alpha }$, the training/test observations (x) under SVM are classified according to the sign of $\hat{f}(x)={\hat{\beta }}_{0}+\overset{N_{S}}{\underset{i=1}{\sum }}{\hat{\alpha }}_{i}y_{i}K\left(x_{i},x\right)$, where ${\hat{\beta }}_{0}=\frac{1}{N_{S}}\underset{i\in S}{\sum }(y_{i}-\underset{j\in S}{\sum }{\hat{\alpha }}_{j}y_{j}K\left(x_{i},x_{j}\right))$ and $N_{S}$ is the total number of support vectors (S) lying on a marginal hyperplane; if f(x)<0, the observation is assigned to the class corresponding to -1, but if f(x)>0, the observation is assigned to the class corresponding to 1 (James *et al.*, 2013).

Also, since most of our data sets contain K > 2 classes in the response variable, we implemented the one-*vs.*-one approach that constructs K(K−1)/2 binary SVMs to compare each pair of classes (k,k’), where one class is coded as +1 and the other as -1. Then, the prediction is done with a voting scheme where a new observation x is assigned to the most frequently assigned class in the K(K−1)/2 binary SVM (James *et al.*, 2013). We implemented the SVM with the radial kernel $K\left(x_{i},x_{i^{’}}\right)=\exp\left[-\gamma \overset{p}{\underset{j=1}{\sum }}{\left(x_{ij}-x_{i^{’}j}\right)}^{2}\right]$, with γ a positive constant (James *et al.*, 2013).The SVM was implemented with the R package e1071 in the R statistical software (R Core Team 2020).

In all models, the relationship matrix G was calculated as $G=\frac{WW^{T}}{\text{q}}$ (as proposed by VanRaden 2008), where W is a matrix of scaled markers (or environmental information) of dimension J×m.The G matrix is a covariance matrix that contains the similarity between individuals based on marker information, pedigree or environmental information. However, for the implementation we obtained the square root of matrix G, and then we post multiplied it for the design matrix of genotypes.

### Maximum a posteriori Threshold (MAPT) genomic prediction model

Instead of using a Gibbs sampler for this model, as done in the BGLR R package, here we propose making predictions with the MAP of the parameters. To do this, an ECM approach is used with the latent variable approach and re-parameterization proposed by Ruud (1991) for obtaining the maximum likelihood estimation. First, note that from the latent representation in model (1), this can be equivalently represented as

$$Y_{i}=c\Leftrightarrow \gamma_{c-1}\leq l_{i}\leq \gamma_{c}\Leftrightarrow 0\leq l_{ic}^{\text{*}}\leq 1$$

where $l_{ic}^{\text{*}}=\frac{l_{i}-\eta_{i}-\gamma_{c-1}}{\gamma_{c}-\gamma_{c-1}}∼N\left[\frac{-\eta_{i}-\gamma_{c-1}}{\left(\gamma_{c}-\gamma_{c-1}\right)},\frac{1}{\left(\gamma_{c}-\gamma_{c-1}\right)}\right]$, for c=2,…,C−1, and $Y_{i}=1\Leftrightarrow l_{i}\leq 0\Leftrightarrow l_{i1}^{\text{*}}=l_{i}-\eta_{i}\leq 0,\;Y_{i}=C\Leftrightarrow l_{i}\geq \gamma_{C}\Leftrightarrow l_{iC}^{\text{*}}=l_{i}-\eta_{i}\geq 0$, where $l_{1}^{\text{*}}∼N\left(-\eta_{i}-\gamma_{1},1\right)$ and $l_{iC}^{\text{*}}∼N\left(-\eta_{i}-\gamma_{C-1},1\right)$. Then, by defining $\delta_{1}=\gamma_{1}$, $\delta_{C}=1$ and $\delta_{c}=\gamma_{c}-\gamma_{c-1}$, c=2,…, C−1, and denoting this modified latent variable as $l_{i}$ instead of $l_{i}^{\text{*}}$, the complete likelihood (based on the observed values $y_{i}$ and the latent variables $l_{i}$) of the parameters is given by

$$L\left(\beta ,\gamma ;y,l\right)=\overset{n}{\underset{i=1}{\prod }}f\left(y_{i},l_{i};\beta ,\gamma \right)=\overset{n}{\underset{i=1}{\prod }}\left\{{\left[f_{l_{i1}^{\text{*}}}\left(l_{i};\beta ,\gamma \right)I_{\left\{-\infty \leq l_{i}\leq 0\right\}}\right]}^{I_{\left\{y_{i}=1\right\}}}\overset{C-1}{\underset{c=2}{\prod }}{\left[f_{l_{ic}^{\text{*}}}\left(l_{i};\beta ,\gamma \right)I_{\left\{0\leq l_{i}\leq 1\right\}}\right]}^{I_{\left\{y_{i}=c\right\}}}{\left[f_{l_{iC}^{\text{*}}}\left(l_{i};\beta ,\gamma \right)I_{\left\{0\leq l_{i}\right\}}\right]}^{I_{\left\{y_{i}=C\right\}}}\right\}$$

and from here the corresponding log-posterior distribution of the parameters is given by

$$\begin{array}{l} \ell_{p}\left(\beta ,\delta ;y,l\right)=\overset{n}{\underset{i=1}{\sum }}\left\{I_{\left\{y_{i}=1\right\}}\left[-\frac{1}{2}\log\left(2\pi \right)-\frac{1}{2}{\left(l_{i}+\eta_{i}+\delta_{1}\right)}^{2}\right]+\overset{C-1}{\underset{c=2}{\sum }}I_{\left\{y_{i}=c\right\}}\left[-\frac{1}{2}\log\left(2\pi \right)+\frac{1}{2}\log\left(\delta_{c}^{2}\right)-\frac{\delta_{c}^{2}}{2}{\left[l_{i}+\left(\eta_{i}+\overset{c-1}{\underset{g=1}{\sum }}\delta_{g}\right)\delta_{c}^{-1}\right]}^{2}\right]+I_{\left\{y_{i}=C\right\}}\left[-\frac{1}{2}\log\left(2\pi \right)-\frac{1}{2}{\left(l_{i}+\eta_{i}+\overset{C-1}{\underset{g=1}{\sum }}\delta_{g}\right)}^{2}\right]\right\}-\frac{1}{2\sigma_{\beta }^{2}}\overset{p}{\underset{j=1}{\sum }}\beta_{j}^{2}-\frac{p}{2}\log\left(\sigma_{\beta }^{2}\right)+\frac{v_{\beta }}{2}\log\left(\frac{S_{\beta }}{2}\right)-\log\left[\text{Γ}\left(\frac{v_{\beta }}{2}\right)\right]-\left(1+\frac{v_{\beta }}{2}\right)\log\left(\sigma_{\beta }^{2}\right)-\frac{S_{\beta }}{2\sigma_{\beta }^{2}}\\ =-\frac{1}{2}\overset{n}{\underset{i=1}{\sum }}\left\{I_{\left\{y_{i}=1\right\}}{\left(l_{i}+\eta_{i}+\delta_{1}\right)}^{2}+\overset{C-1}{\underset{c=2}{\sum }}I_{\left\{y_{i}=c\right\}}\left[-\log\left(\delta_{c}^{2}\right)+{\left[l_{i}\delta_{c}+\left(\eta_{i}+\overset{c-1}{\underset{g=1}{\sum }}\delta_{g}\right)\right]}^{2}\right]+I_{\left\{y_{i}=C\right\}}{\left(l_{i}+\eta_{i}+\overset{C-1}{\underset{g=1}{\sum }}\delta_{g}\right)}^{2}\right\}-\frac{1}{2\sigma_{\beta }^{2}}\overset{p}{\underset{j=1}{\sum }}\beta_{j}^{2}-\frac{p}{2}\log\left(\sigma_{\beta }^{2}\right)+\frac{v_{\beta }}{2}\log\left(\frac{S_{\beta }}{2}\right)-\log\left[\text{Γ}\left(\frac{v_{\beta }}{2}\right)\right]-\left(1+\frac{v_{\beta }}{2}\right)\log\left(\sigma_{\beta }^{2}\right)-\frac{S_{\beta }}{2\sigma_{\beta }^{2}} \end{array}$$

where $\delta =\left(\delta_{1},\ldots ,\delta_{C-1}\right).$ The expected value of this complete log-posterior with respect to the conditional distribution of the latent variables l given the observations y, and current values of the parameters, $\beta^{\left(t\right)}$ and $\delta^{\left(t\right)}$, is given by

E-Step (a):

$$Q\left(\beta ,\delta |\beta^{\left(t\right)},\;\delta^{\left(t\right)}\right)=E\left[\ell_{p}\left(\beta ,\delta ;y,l\right)|\beta^{\left(t\right)},\;\delta^{\left(t\right)},y\right]=-\frac{1}{2}\overset{n}{\underset{i=1}{\sum }}\left\{I_{\left\{y_{i}=1\right\}}\left[l_{i}^{\text{**}}+{\left(l_{i}^{\text{*}}+\eta_{i}+\delta_{1}\right)}^{2}\right]+\overset{C-1}{\underset{c=2}{\sum }}I_{\left\{y_{i}=c\right\}}\left(-2\log\left(\delta_{c}\right)+\left\{l_{i}^{\text{**}}\delta_{c}^{2}+{\left[l_{i}^{\text{*}}\delta_{c}+\eta_{i}+\overset{c-1}{\underset{g=1}{\sum }}\delta_{g}\right]}^{2}\right\}\right)+I_{\left\{y_{i}=C\right\}}\left[l_{i}^{\text{**}}+{\left(l_{i}^{\text{*}}+\eta_{i}+\overset{C-1}{\underset{g=1}{\sum }}\delta_{g}\right)}^{2}\right]\right\}$$

where $l_{i}^{\text{*}}=\text{E}\left[l_{i}|\beta^{\left(t\right)},\;\delta^{\left(t\right)},y_{i}\right]$ and $l_{i}^{\text{**}}=\text{Var}\left[l_{i}|\beta^{\left(t\right)},\;\delta^{\left(t\right)},y_{i}\right]$ are the mean and variance of the conditional distribution of the latent variable $l_{i}$. For $y_{i}=c$, c=2,…,C−1, $l_{i}|\beta^{\left(t\right)},\;\delta^{\left(t\right)},y_{i}$ is a truncated normal distribution in (0,1) with mean $-(\eta_{i}+\overset{c-1}{\underset{g=1}{\sum }}\delta_{g})/\delta_{c}$ and variance $1/\delta_{c}$, while for $y_{i}=1$, $l_{i}|\beta^{\left(t\right)},\;\delta^{\left(t\right)},y_{i}$ is a truncated normal distribution in $\left(-\infty ,\delta_{1}\right)$ with mean $-(\eta_{i}+\delta_{1})$ and variance 1, and when $y_{i}=C$, $l_{i}|\beta^{\left(t\right)},\;\delta^{\left(t\right)},y_{i}$ is also a truncated normal distribution in (0,∞) with mean $-\left(\eta_{i}+\overset{C-1}{\underset{g=1}{\sum }}\delta_{g}\right)$ and variance 1 (in the implementation this was computed with the R package truncnorm, Mersmann *et al.*, 2018).

In the ECM algorithm, the M-step in the EM is replaced by several computationally simpler conditional maximization (CM)-steps, where in each of these steps, $Q\left(\beta ,\delta |\beta^{\left(t\right)},\;\delta^{\left(t\right)}\right)$ is maximized with respect to one parameter at a time, keeping the others fixed, and repeating this for each parameter. Specifically, the CM-step in this model is given by:

CM-steps (b):

Step 1: $\delta_{1}^{\left(t+1\right)}=-\frac{1}{n}\overset{n}{\underset{i=1}{\sum }}\left\{\left(l_{i}^{\text{*}}+\eta_{i}\right)I_{\left\{y_{i}=1\right\}}+I_{\left\{y_{i}=2\right\}}\left(l_{i}^{\text{*}}\delta_{2}+\eta_{i}\right)+\overset{C}{\underset{c=3}{\sum }}I_{\left\{y_{i}=c\right\}}\left[l_{i}^{\text{*}}\delta_{c}+\eta_{i}+\overset{c-1}{\underset{g=2}{\sum }}\delta_{g}\right]\right\}$.
Step 2: For k=2,…,C−1, $\delta_{k}^{\left(t+1\right)}$ is the positive solution of the following quadratic equation:
  $$-\overset{n}{\underset{i=1}{\sum }}I_{\left\{y_{i}=k\right\}}+\left(\overset{n}{\underset{i=1}{\sum }}I_{\left\{y_{i}=k\right\}}l_{i}^{\text{*}}\eta_{i}+\left(\overset{n}{\underset{i=1}{\sum }}I_{\left\{y_{i}=k\right\}}l_{i}^{\text{*}}\right)\overset{k-1}{\underset{g=1}{\sum }}\delta_{g}+\overset{C}{\underset{c=k+1}{\sum }}\left(\overset{n}{\underset{i=1}{\sum }}I_{\left\{y_{i}=c\right\}}\left(l_{i}^{\text{*}}\delta_{c}+\eta_{i}\right)+\left(\overset{n}{\underset{i=1}{\sum }}I_{\left\{y_{i}=c\right\}}\right)\overset{c-1}{\underset{\begin{array}{c} g=1\\ g\neq k \end{array}}{\sum }}\delta_{g}\right)\right)\delta_{k}+\left(\overset{n}{\underset{i=1}{\sum }}I_{\left\{y_{i}=k\right\}}\left(l_{i}^{\text{**}}+l_{i}^{2\text{*}}\right)+\overset{C}{\underset{c=k+1}{\sum }}\overset{n}{\underset{i=1}{\sum }}I_{\left\{y_{i}=c\right\}}\right)\delta_{k}^{2}=0.$$
Step 3: For $j=1,\ldots ,p,\;\beta_{j}^{\left(t+1\right)}=-\frac{\sum_{i=1}^{n}\left\{x_{ij}\left(l_{i}^{\text{*}}+\eta_{ij}+\delta_{1}\right)I_{\left\{y_{i}=1\right\}}+\sum_{c=2}^{C}I_{\left\{y_{i}=c\right\}}x_{ij}\left(l_{i}^{\text{*}}\delta_{c}+\eta_{ij}+\sum_{g=1}^{c-1}\delta_{g}\right)\right\}}{\sum_{i=1}^{n}x_{ij}^{2}+\sigma_{\beta }^{-2}}$, where $\eta_{ij}=\overset{p}{\underset{\begin{array}{c} k=1\\ k\neq j \end{array}}{\sum }}x_{ik}\beta_{k}$.
Step 4: $\sigma_{\beta }^{2}{}^{\left(t+1\right)}=\frac{\frac{1}{2}\left(S_{\beta }+\beta^{T}\beta \right)}{1+\frac{v_{\beta }}{2}+\frac{p}{2}}$.

These steps were obtained by solving the derivative of $Q\left(\beta ,\delta |\beta^{\left(t\right)},\;\delta^{\left(t\right)}\right)$ with respect to each parameter equal to 0. For example, for k=2,…,C−1, the derivative of this quantity with respect to $\delta_{k}$, and equal to 0 is given by

$$\frac{\partial }{\partial \delta_{k}}Q\left(\beta ,\delta |\beta^{\left(t\right)},\;\delta^{\left(t\right)}\right)=-\overset{n}{\underset{i=1}{\sum }}\left\{I_{\left\{y_{i}=k\right\}}\left[-\frac{1}{\delta_{k}}+l_{i}^{\text{**}}\delta_{k}+\left(l_{i}^{\text{*}}\delta_{k}+\eta_{i}+\overset{k-1}{\underset{g=1}{\sum }}\delta_{g}\right)l_{i}^{\text{*}}\right]\right\}-\overset{n}{\underset{i=1}{\sum }}\left\{\overset{C}{\underset{c=k+1}{\sum }}I_{\left\{y_{i}=c\right\}}\left(l_{i}^{\text{*}}\delta_{c}+\eta_{i}+\overset{c-1}{\underset{g=1}{\sum }}\delta_{g}\right)\right\}=0.$$

Then, by multiplying by $\delta_{k}$ and grouping terms, step 2 is obtained. This is similar for the rest of the parameters. In all the CM-steps, the updated parameters obtained in the above CM-steps are used.

When a flat prior is assumed for the beta coefficients, step 4 is removed, and step 3 of the CM-steps is replaced by

$$\beta_{j}^{\left(t+1\right)}=-\frac{\sum_{i=1}^{n}\left\{x_{ij}\left(l_{i}^{\text{*}}+\eta_{ij}+\delta_{1}\right)I_{\left\{y_{i}=1\right\}}+\sum_{c=2}^{C}I_{\left\{y_{i}=c\right\}}x_{ij}\left(l_{i}^{\text{*}}\delta_{c}+\eta_{ij}+\sum_{g=1}^{c-1}\delta_{g}\right)\right\}}{\sum_{i=1}^{n}x_{ij}^{2}}.$$

The extension of ECM implementation occurs almost immediately when more predictors are included in this model.

### Hyperparameter specification

Hyperparameter specification in genomic prediction is very important for building models with reasonable prediction performance. Here, we adopted the strategy used in the BGLR package (Pérez and de los Campos 2014), but with some modifications. We assigned a proportion $1-R^{2}$of the total variability to the latent variable, to the linear predictor $-x_{i}^{T}\beta$. Because the average variance of the latent variables across the individuals is equal to

$$V_{l}=\frac{1}{n}\overset{n}{\underset{i=1}{\sum }}Var\left(l_{i}\right)=\frac{1}{n}tr\left(XX^{T}\right)\sigma_{\beta }^{2}+1$$

then by fixing a value for $v_{\beta }$, the prior average of the proportion of the total variability explained by the linear predictor is $1-R^{2}$ when the scale parameter of the prior distribution of the variance of the beta coefficients is chosen to be $S_{\beta }=\frac{\left(1-R^{2}\right)V_{l}}{\frac{1}{n}tr\left(XX^{T}\right)}\left(v_{\beta }-2\right)$. We used $R^{2}=0.5\;$and the $v_{\beta }$ value used in the applications was set by default to 1000, which induced a prior distribution for $\sigma_{\beta }^{2}$ with mean $\frac{\left(1-R^{2}\right)V_{l}}{\frac{1}{n}tr\left(XX^{T}\right)}$ and a coefficient of variation of about 4.48%.

### Phenotypic data sets

We used 8 data sets, of which data sets 1-7 were used by Montesinos-López *et al.* (2019) and data set 8 was used by Montesinos-López *et al.* (2015). More specific details of these data sets can be found in these articles: Montesinos-López *et al.*, 2015 and 2019. Data sets 1-7 belong to four elite yield trial (EYT) nurseries from the Global Wheat Program of the International Maize and Wheat Improvement Center (CIMMYT), that were evaluated at the Norman E. Borlaug Research Station, Ciudad Obregon, Sonora, Mexico. All these nurseries were evaluated during four seasons: 2013-2014 (EYT 13-14; here called data set 1 with 767 lines), 2014-2015 (EYT 14-15; called data set 2 with 775 lines), 2015-2016 (EYT 15-16; called data set 3 with 964 lines) and 2016-2017 (EYT 16-17; called data set 4 with 980 lines). Most of these data sets were evaluated in the following six environments: Bed2IR, Bed5IR, Flat5IR, FlatDrip, EHT and LHT. In this publication we used only the information of two discretized traits, Days to Heading (DTHD) and days to maturity (DTMT), with five levels each (1, 2, 3, 4, 5).

Data set 5 is part of data set 3; for this reason, the phenotypic information and genomic information were obtained in the same way as in data set 3. However, only 964 lines of the total 980 lines under study in data set 3 had complete data. But now we used three traits measured in data set 5: grain color (GC) (1 = yes, 2 = no), leaf rust (ordinal scale with 5 points) and stripe rust (ordinal scale with 3 points). Data set 6 and data set 7 are part of the wheat yield trial (YT) nurseries from CIMMYT’s Global Wheat Breeding Program. For data set 6, the number of lines used was 945, and for data set 7, 1145 wheat lines were used. In this publication we only used the ordinal trait lodging (ordinal scale of 5 points) measured on both data sets.

Data set 8 contains information of 278 maize lines on Gray Leaf Spot (GLS) disease which is caused by the fungus *Cercospora zeae-maydis*. This data set contains phenotypic and genotypic information of the 278 maize lines from the Drought Tolerance Maize (DTMA) project of CIMMYT’s Global Maize Program. The data set was originally analyzed by Crossa *et al.* (2011), and re-analyzed later by González-Camacho *et al.* (2012), Montesinos-López *et al.* (2015) and Pérez-Rodríguez *et al.* (2018) using different statistical models. The data set includes information on disease severity measured on an ordinal scale with 5 points: 1 = no disease, 2 = low infection, 3 = moderate infection, 4 = high infection and 5 = totally infected.

### Genotypic data

Data sets 1, 2, 3, and 4 were genotyped using genotyping-by-sequencing (GBS) (Elshire *et al.*, 2011; Poland *et al.*, 2012) at Kansas State University, using an Illumina HiSeq2500 for obtaining genome-wide markers. Marker polymorphisms were called across all lines using the TASSEL (Trait Analysis by Association Evolution and Linkage) GBS pipeline (Glaubitz *et al.*, 2014) and anchored to the International Wheat Genome Sequencing Consortium’s (IWGSC) first version of the reference sequence (RefSeq v1.0) assembly of the bread wheat variety Chinese Spring. Markers with more than 60% missing data, less than 5% minor allele frequency and percent heterozygosity greater than 10% were removed; as a result, we obtained 2,038 markers. Missing marker data were imputed using LinkImpute (Money *et al.*, 2015) implemented in TASSEL (Bradbury *et al.*, 2007), version 5. The lines under study were filtered for more than 50% missing data and we ended up with 3,486 lines (79.807%) of the total 4,368 lines originally evaluated in four seasons (767 lines from data set 1, 775 lines from data set 2, 964 lines from data set 3 and 980 lines from data set 4) (Juliana *et al.*, 2018). The lines used in data sets 5, 6, and 7 were genotyped with the same marker system that was used for the other data sets.

The lines of data set 8 were initially genotyped with 1,152 SNPs and re-genotyped later with 55k SNPs using the Illumina platform. After removing SNPs with more than 10% missing values and imputing filtering markers with minor allele frequency smaller than 0.05, a total of 46,347 markers were still available for further analysis. The data set containing the phenotypic and genotypic information can be downloaded from http://hdl.handle.net/11529/10254.

### Data availability

Details of the phenotypic and genomic data of the first seven data sets used in this study can be downloaded from the link: http://hdl.handle.net/11529/10548140. Data set 8 is available at http://hdl.handle.net/11529/10254.

### Metrics used to measure prediction performance

To evaluate the prediction performance, we used a type of cross-validation that mimics a situation where lines were evaluated in some environments for all traits but where some lines were missing in other environments. We implemented a fivefold cross-validation, using four folds for training and one for testing. We reported the average of the five folds of the proportion of cases correctly classified (PCCC). It is important to point out that the process for tuning the hyper-parameter (λ) in the multinomial Ridge regression was done with ten-fold cross-validation. Also, for the PCCC we computed the standard error (SE) in each fold using 500 bootstrap samples (of observed and predicted values from the testing); then the average of the 5 SE was reported as a measure of variability in each metric. It is important to point out that the fivefold cross-validation strategy was implemented with only 1 replication.

## Results

The results are given in seven main sections. Each section provides the prediction performance of each data set with the proposed methods, except that data sets 6 and 7 are given in the same section. In each section the proposed method (M2) is compared with the multinomial ridge regression (M3) model, support vector machine (M4), and the Bayesian threshold genomic best linear unbiased prediction model (M1).

### Data set 1

In this data set, the levels of both response variables were five (1, ...,5). It is important to point out that here the predictor contains information with (E+G+GE) and without the genotype by interaction term (E+G); E refers to environment information, G refers to the genotypes incorporating the genomic relationship information and GE refers to the genotype by environment interaction. First, we compared the prediction performance in trait DTHD of the proposed MAPT (model M2) algorithm with the Bayesian Threshold Genomic prediction model (model M1) implemented in the BGLR package, the classic Multinomial Ridge regression (model M3) implemented in the library glmnet, and the support vector machine (model M4) implemented in the library e1071. Then, we did the same for trait DTMT. The panels (right and left) in all figures except Figures 9 and 10 give the results of the models with and without interaction.

**Figure 1:**
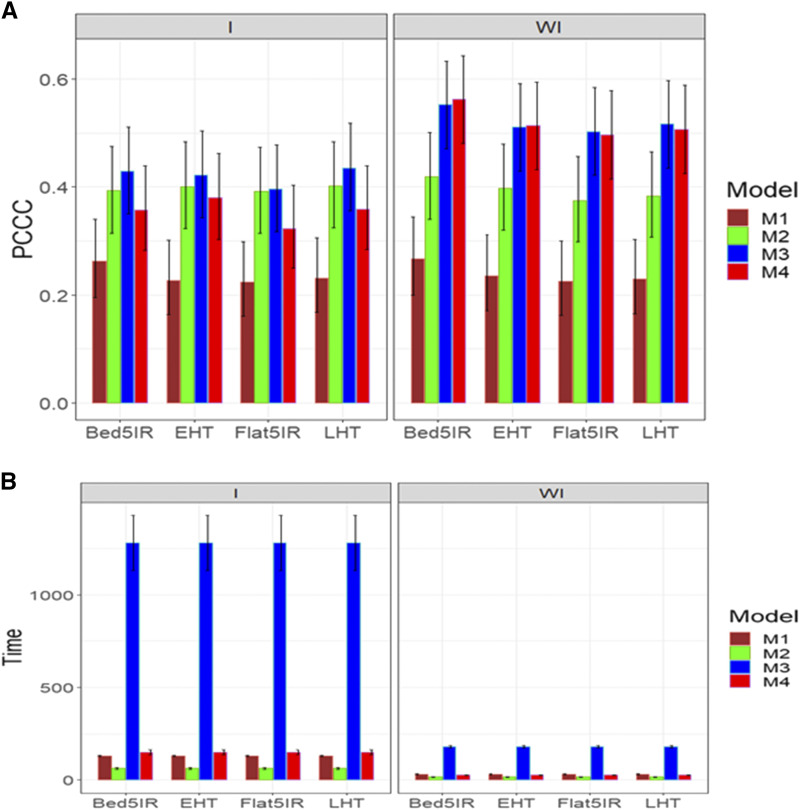
Average prediction performance in terms (A) of the proportion of cases correctly classified (PCCC), and (B) the implementation time in minutes (Time) of the four models (M1= Bayesian threshold genomic best linear unbiased prediction model in BGLR, M2 = MAPT model, M3 = multinomial model in glmnet and M4= support vector machine) for data set 1 in trait DTHD. The left panel is with interaction (I) and the right panel is without interaction (WI).

Figure 1A shows that in general the best predictions for trait DTHD were observed when the genotype by environment interaction was ignored under models M3 and M4; however, models M3 and M4 were not statistically better than model M2. The worst performance was observed under model M1 with and without taking into account the genotype by environment interaction term (Figure 1A). Also, without genotype by environment interaction, no statistical differences were observed in the prediction performance of models M2, M3 and M4, which outperformed model M1 in most environments. With regard to the implementation time, Figure 1B indicates that when ignoring the genotype by environment interaction, the best models were M2 and M4 and the slowest was model M3. When taking into account the interaction term, model M2 was the best in implementation time, while the worst was model M3 (Figure 1B; right panel). Also, the largest gain in terms of time of performance of M2 compared to the other models was observed when considering the genotype by environment interaction.

**Figure 2:**
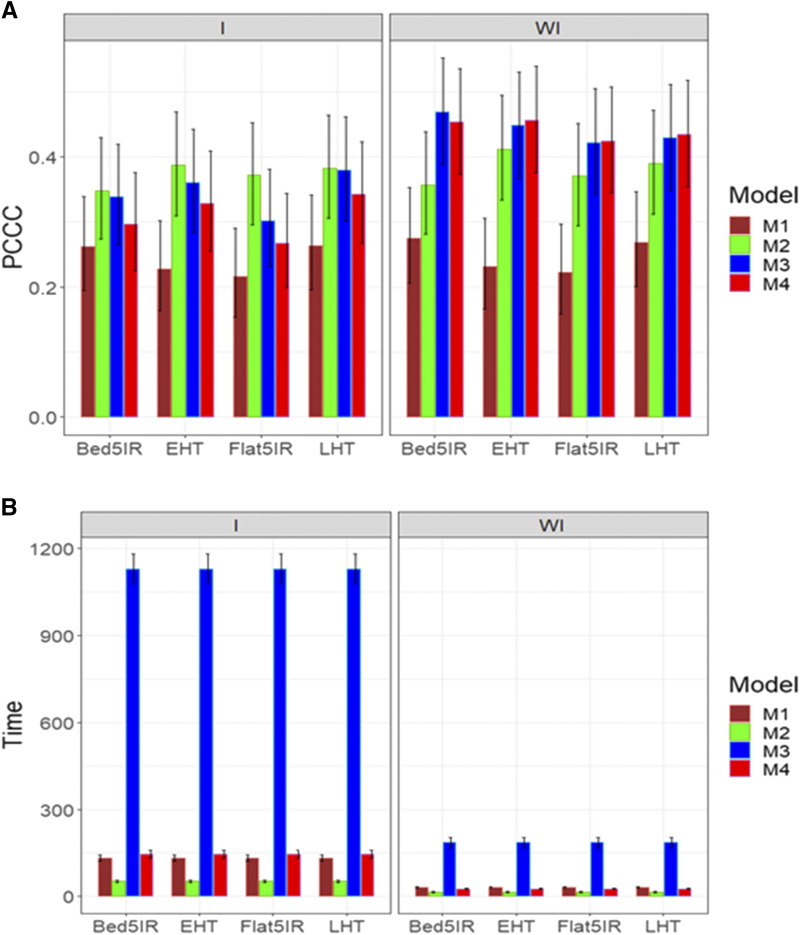
Average prediction performance in terms (A) of the proportion of cases correctly classified (PCCC) and (B) the implementation time in minutes (Time) of the four models (M1= Bayesian threshold genomic best linear unbiased prediction model in BGLR, M2 = MAPT model, M3 = multinomial model in glmnet and M4= support vector machine) for data set 1 in trait DTMT. The left panel is with interaction (I) and the right panel is without interaction (WI).

For trait DTMT, we also obtained the best predictions when ignoring the genotype by environment interaction with models M3 and M4, although they were not statistically better than the proposed model M2 (Figure 2A). In general, the worst performance in terms of prediction was observed in model M1. Taking into account the genotype by environment interaction, we observed (Figure 2A) in the four environments that model M2 was the best in terms of prediction performance but was not statistically better than models M3 and M4 and was better than model M1 in two out of the four environments. Regarding the implementation time, without taking into account the genotype by environment interaction, Figure 2B **(**right panel) indicates that the slowest model was model M3 and the fastest was model M2; however, a large difference was not observed between the time required for models M2 and M4. On the other hand, taking into account the genotype by environment interaction, the shortest implementation time was observed in model M2 and the slowest in model M3 (Figure 2B; left panel), and taking into account the genotype by environment interaction, model M2 showed the greatest superiority in terms of implementation time compared to the other models (Figure 2B).

### Data set 2

**Figure 3:**
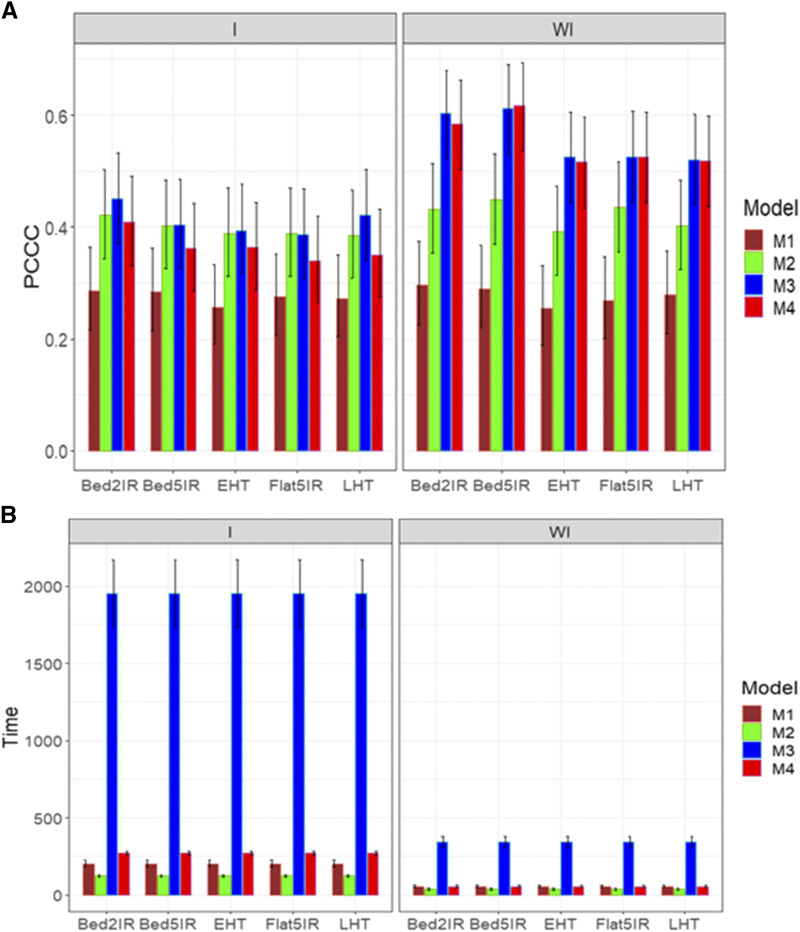
Average prediction performance in terms (A) of the proportion of cases correctly classified (PCCC) and (B) the implementation time in minutes (Time) of the four models (M1= Bayesian threshold genomic best linear unbiased prediction model in BGLR, M2 = MAPT model, M3 = multinomial model in glmnet and M4= support vector machine) for data set 2 in trait DTHD. The left panel is with interaction (I) and the right panel is without interaction (WI).

In data set 2, there were five (1, ..., 5) levels of the response variable. In this data set, the predictor contains information with (E+G+GE) and without the genotype by interaction term (E+G). Figure 3A gives the prediction performance for trait DTHD. Here also the best predictions were observed when ignoring the genotype by environment interaction under models M3 and M4, but in most cases, these models were not statistically better than model M2. In general, the worst prediction performance was observed under model M1, but in most cases it was not statistically different than model M2 (Figure 3A). Taking into account the genotype by environment interaction, the prediction performances of M2, M3 and M4 were very similar (no statistical differences were found). With regard to implementation time, in all environments the best time was in model M2 and the slowest in model M3; however, the gain in implementation time of M2 compared to the other models was less when the genotype by environment interaction term was not taken into account (Figure 3B; right panel).

**Figure 4:**
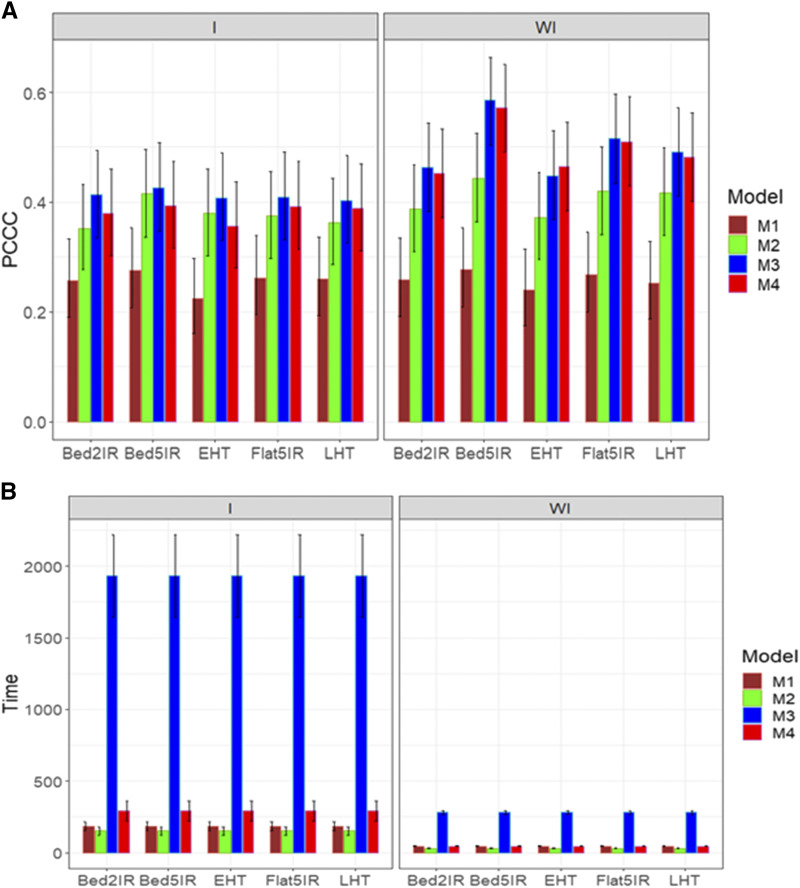
Average prediction performance in terms (A) of the proportion of cases correctly classified (PCCC) and (B) the implementation time in minutes (Time) of the four models (M1= Bayesian threshold genomic best linear unbiased prediction model model in BGLR, M2 = MAPT model, M3 = multinomial model in glmnet and M4= support vector machine) for data set 2 in trait DTMT. The left panel is with interaction (I) and the right panel is without interaction (WI).

For trait DTMT, the best performance in terms of PCCC was observed when the genotype by environment interaction term was ignored and, again, models M3 and M4 were the best in terms of prediction performance, but in all cases no statistical differences were observed with regard to model M2 (Figure 4A). In general, M1 had the worst prediction performance. When the genotype by environment interaction was taken into account, the differences between models M2, M3 and M4 were smaller, but under this scenario, many times model M1 was not statistically different from model M2 (Figure 4A). With regard to implementation time, the fastest models were models M1 and M2 (taking into account the interaction term), but models M1, M2 and M4 were the slowest when the genotype by environment interaction term was ignored; however, the implementation time for model M3 is very expensive compared to the other 3 models (Figure 4B).

### Data set 3

**Figure 5:**
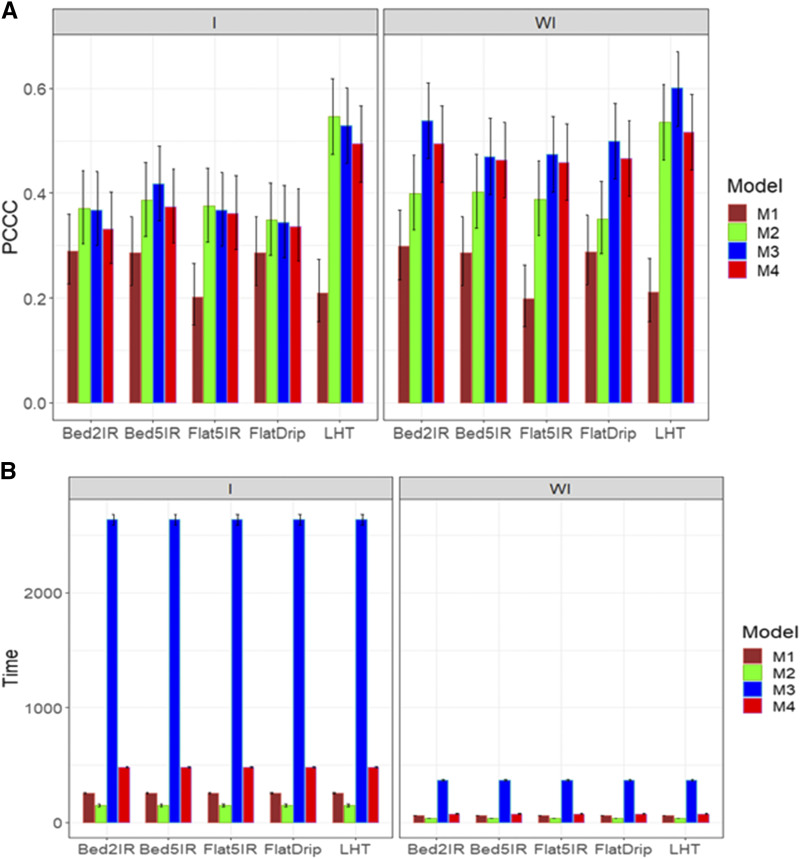
Average prediction performance in terms (A) of the proportion of cases correctly classified (PCCC) and (B) the implementation time in minutes (Time) of the four models (M1= Bayesian threshold genomic best linear unbiased prediction model in BGLR, M2 = MAPT model, M3 = multinomial model in glmnet and M4= support vector machine) for data set 3 in trait DTHD. The left panel is with interaction (I) and the right panel is without interaction (WI).

First, we explain the prediction performance of the 4 models for trait DTHD. The same predictor as in data sets 1 and 2 was used with (E+G+GE) and without the genotype by interaction term (E+G). Figure 5A indicates that when the genotype by environment interaction was not taken into account, the best models were models M3, M4 and M2. However, no statistical differences were observed between these three models in terms of prediction performance, but in general the worst model was model M1 (Figure 5A). When the genotype by environment interaction was taken into account, model M2 was the best in four of the five environments; however, it was not statistically superior to models M3 and M4. In general, the best predictions were obtained when the genotype by environment interaction was ignored. It is important to point out that in environment LHT, the best predictions occurred under models M2-M4, but generally in all environments, the predictions were larger than random guessing (20% since the response variable has five levels). With regard to the implementation time, the best model was model M2, but the gain was larger for this model compared to the other three models when genotype by environment interaction was taken into account (Figure 5B; left panel). It is important to point out that the slowest time performance was observed in model M3, that is, it was many times longer than the time performance of the other models (Figure 5B).

**Figure 6:**
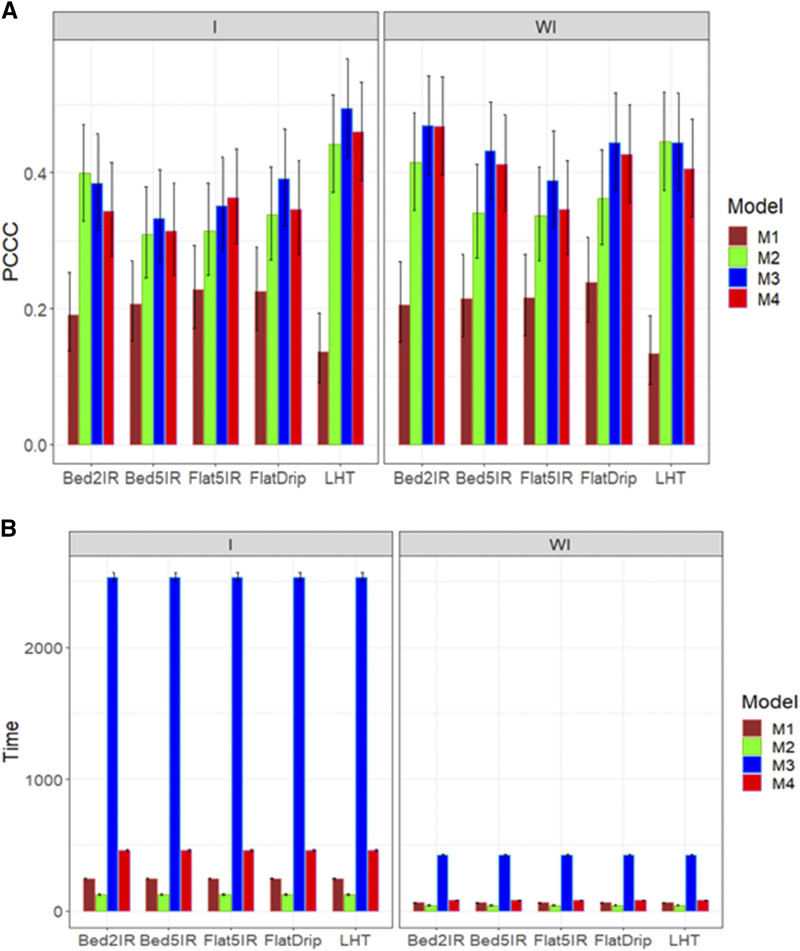
Average prediction performance in terms (A) of the proportion of cases correctly classified (PCCC) and (B) the implementation time in minutes (Time) of the four models (M1= Bayesian threshold genomic best linear unbiased prediction model in BGLR, M2 = MAPT model, M3 = multinomial model in glmnet and M4= support vector machine) for data set 3 in trait DTMT. The left panel is with interaction (I) and the right panel is without interaction (WI).

For trait DTMT, the best prediction performance was observed when the genotype by environment interaction was ignored, and again the best predictions were observed under model M3 and the worst under model M1. However, model M3 was not statistically better than models M2 and M4. When the genotype by environment interaction was considered, models M2, M3 and M4 were the best and M1 was the worst (Figure 6A). In terms of implementation time, model M2 was the best and model M3 the slowest, and again the major gain in terms of implementation time was observed in models with genotype by environment interaction (Figure 6B; left panel).

### Data set 4

**Figure 7:**
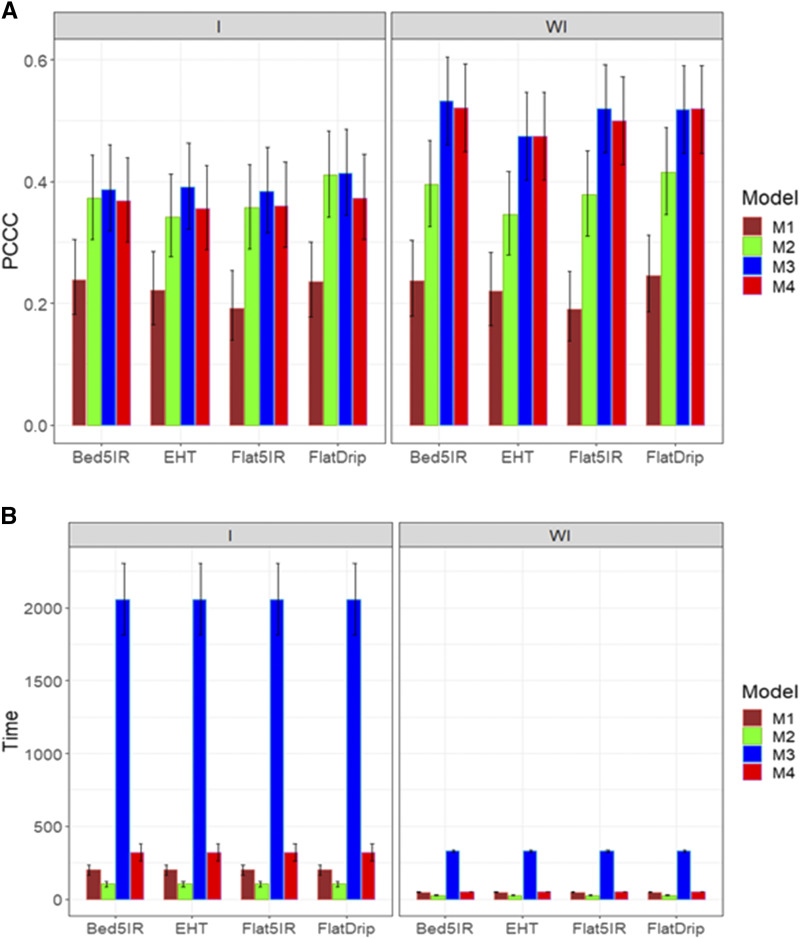
Average prediction performance in terms (A) of the proportion of cases correctly classified (PCCC) and (B) the implementation time in minutes (Time) of the four models (M1= Bayesian threshold genomic best linear unbiased prediction model in BGLR, M2 = MAPT model, M3 = multinomial model in glmnet and M4= support vector machine) for data set 4 in trait DTHD. The left panel is with interaction (I) and the right panel is without interaction (WI).

The same two predictors as in the previous three data sets were used with (E+G+GE) and without the genotype by interaction term (E+G). For trait DTHD, Figure 7A shows that when the genotype by environment interaction was ignored, the best models in terms of PCCC were models M3 and M4 and the worst was model M1. When the genotype by environment interaction was taken into account, models M2, M3 and M4 were the best and the worst again was model M1. However, in general, the best predictions were observed when the genotype by environment interaction was ignored (Figure 7A). The prediction performance was quite similar across environments for each model (Figure 7A). As for the implementation time, again the best model was model M2 and the worst was model M3. However, under genotype by environment interaction, the largest gain was observed with model M2 compared to the other models (Figure 7B; left panel). It is important to point out that, in general, model M3 was many times slower in terms of implementation time than the other models.

**Figure 8:**
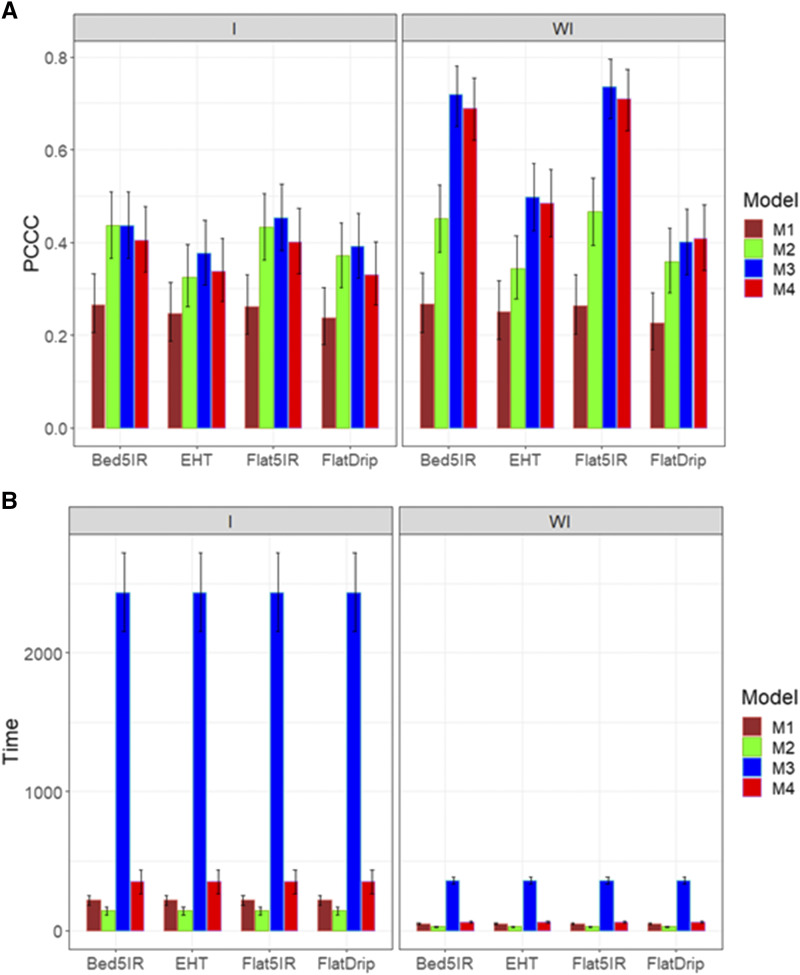
Average prediction performance in terms (A) of the proportion of cases correctly classified (PCCC) and (B) the implementation time in minutes (Time) of the four models (M1= Bayesian threshold genomic best linear unbiased prediction model in BGLR, M2 = MAPT model, M3 = multinomial model in glmnet and M4= support vector machine) for data set 4 in trait DTMT. The left panel is with interaction (I) and the right panel is without interaction (WI).

For trait DTMT, when the genotype by environment interaction was not taken into account, models M3 and M4 were the best in terms of PCCC, and model M1 was the worst (Figure 8A; right panel). However, when the genotype by environment interaction was considered, models M2, M3 and M4 were the best and model M1 was the worst (Figure 8A; left panel). The best predictions were observed without considering the genotype by environment interaction and the predictions of environments Bed5IR and Flat5IR were considerably better with models M3 and M4 (Figure 8A; right panel). With regard to the implementation time, again the best model was model M2 and the slowest model was M3, but again the largest gain in terms of implementation time was observed when genotype by environment interaction was taken into account (Figure 8B; right panel). In both scenarios, with and without the genotype by environment interaction, the best model in terms of implementation time was M2, the second best was model M1, the third best was model M4 and the worst was model M3.

### Data set 5

**Figure 9:**
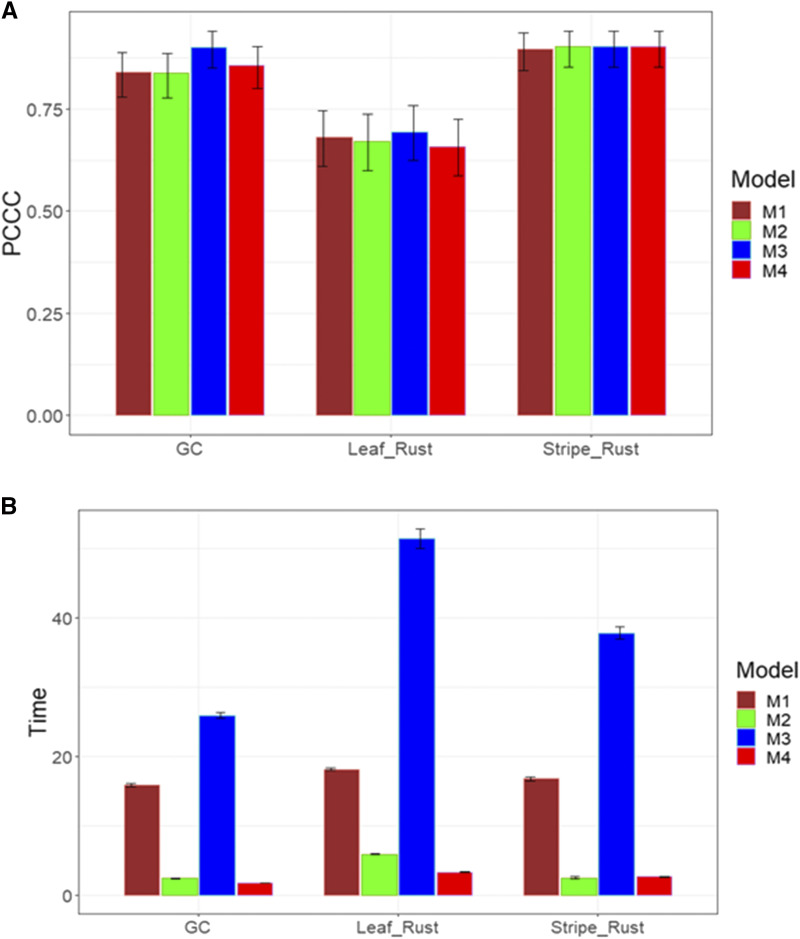
Average prediction performance in terms (A) of the proportion of cases correctly classified (PCCC) and (B) the implementation time in minutes (Time) of the four models (M1= Bayesian threshold genomic best linear unbiased prediction model in BGLR, M2 = MAPT model, M3 = multinomial model in glmnet and M4= support vector machine) for data set 5 in traits GC, Leaf_Rust and Stripe_Rust.

This data set contains three ordinal traits; for this reason, we report the prediction performance for trait GC (binary trait), Leaf_Rust (5 levels) and Stripe_Rust (3 levels). The predictor now only contains information on genotypes (G). For traits GC, Leaf_Rust and Stripe_Rust, we found no statistical differences between the four models in terms of prediction performance, but the best predictions were observed in trait Stripe_Rust and the worst in trait Leaf_Rust (Figure 9A). In terms of implementation time, we found in all traits that the best time was observed in models M2 and M4, but the worst time was in model M3 (Figure 9B). However, the longest implementation time was observed for trait Leaf_Rust and the shortest for trait GC (Figure 9B).

### Data set 6-7

**Figure 10:**
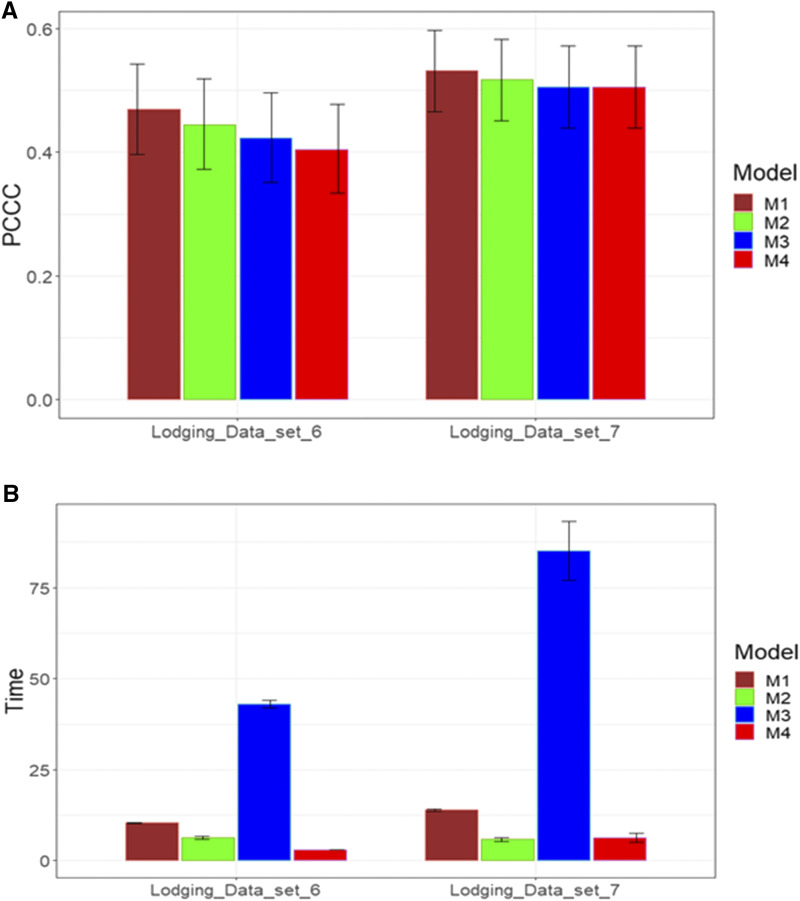
Average prediction performance in terms (A) of the proportion of cases correctly classified (PCCC) and (B) the implementation time in minutes (Time) of the four models (M1= Bayesian threshold genomic best linear unbiased prediction model in BGLR, M2 = MAPT model, M3 = multinomial model in glmnet and M4= support vector machine) for data sets 6 and 7 in trait Lodging.

Both these data sets have only one trait (lodging) with five levels in the response variable. The predictor in these two data sets only contains information on genotypes (G). In terms of prediction performance, in both data sets we found no statistical differences between the four models even though models M1 and M2 were slightly better. The prediction performance in data set 7 was much better than in data set 6 (Figure 10A). With regard to the implementation time, in data set 6 the best performance was observed under model M4, followed by model M2, and the worst performance occurred under model M3 (Figure 10B). In data set 7, the best implementation time was observed in models M2 and M4 and the worst again in model M3 (Figure 10B). In general, the implementation time was longer in data set 7 than in data set 6 (Figure 10B). Finally, in both data sets, the implementation time of model M3 was many times longer than the time of the other models (Figure 10B).

### Data set 8

**Figure 11:**
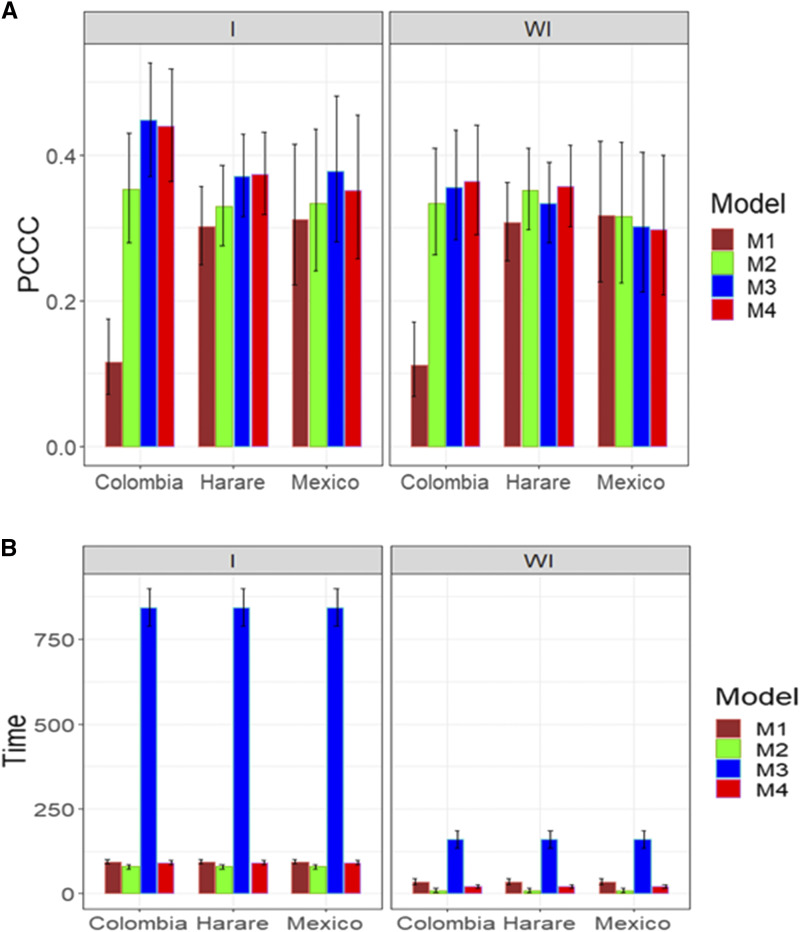
Average prediction performance in terms (A) of the proportion of cases correctly classified (PCCC) and (B) the implementation time in minutes (Time) of the four models (M1= Bayesian threshold genomic best linear unbiased prediction model in BGLR, M2 = MAPT model, M3 = multinomial model in glmnet and M4= support vector machine) for data set 8 in trait GLS with a simple predictor. The left panel is with interaction (I) and the right panel is without interaction (WI).

In this data set, the only trait evaluated was GLS with five levels. Figure 11A gives the prediction performance with the simple predictor with interaction (E + G + GE + A + AE) and without interaction (E + G + A; A refers to the genotypes incorporating pedigree information). In one out of three environments, models M2, M3 and M4 outperformed model M1. A similar pattern was observed with and without the genotype by environment interaction, but taking into account the genotype by environment interaction was slightly better than ignoring it (Figure 11A). With regard to the implementation time, in general, the best performance was observed in model M2, then in model M4 and then in model M1, and the worst performance was observed in model M3 (Figure 11B). The implementation time was considerably longer when the genotype by environment interaction was taken into account (Figure 11B) and model M3 was considerably slower in terms of implementation time than the other models (Figure 11B).

**Figure 12:**
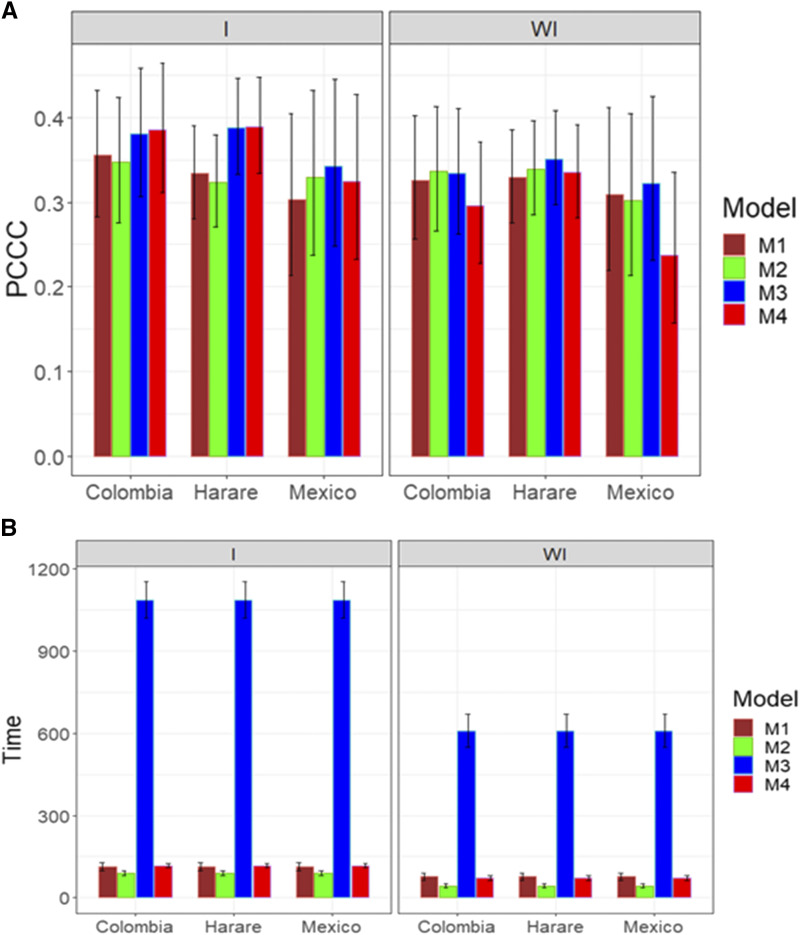
Average prediction performance in terms (A) of the proportion of cases correctly classified (PCCC) and (B) the implementation time in minutes (Time) of the four models (M1= Bayesian threshold genomic best linear unbiased prediction model in BGLR, M2 = MAPT model, M3 = multinomial model in glmnet and M4= support vector machine) for data set 8 in trait GLS with a more complex predictor (with Rep). The left panel is with interaction (I) and the right panel is without interaction (WI).

The prediction performance with the more complex predictor (with interaction: E + G + GE + A + AE + Rep + ERep + GRep + ARep, and without interaction: E + G + A + Rep + ERep + GRep + ARep; Rep refers to the effects of replications, ERep to the interaction between the environment and replications, GRep to the interaction between the genotypes and replications and ARep to the interaction between the lines with pedigree and replications) for the GLS trait is given in Figure 12. Figure 12A shows no statistical differences in terms of prediction performance between the four models with and without the genotype by environment interaction term. However, in general, the performance was better taking into account the genotype by environment interaction (Figure 12A). On the other hand, with regard to the implementation time, model M2 was the best, model M4 the second best and model M1 the third best, while model M3 was the worst. The required implementation time was many times longer in model M3 compared to the other models (Figure 12B).

### Sensitivity of the priors

In this section we evaluated the degree of sensitivity of the priors in the prediction performance of the proposed MAPT model. As mentioned in material and methods, the prior of the beta coefficients is informative and the degree of informativeness depends on the coefficient of variation of the prior distribution of $\sigma_{\beta }^{2}$ which is equal to CV=2(vβ−4), that does not depend on the scale parameter. For large values of $v_{\beta }$, this prior will concentrate around its mean $\frac{S_{\beta }}{v_{\beta }-2}$. Therefore, according to the hyper-parameter specification described before (in material and methods) $S_{\beta }=\frac{\left(1-R^{2}\right)V_{l}}{\frac{1}{n}tr\left(XX^{T}\right)}\left(v_{\beta }-2\right)$, and this indicates that its mean is reduced to

$$E\left(\sigma_{\beta }^{2}\right)=\frac{S_{\beta }}{v_{\beta }-2}=\frac{\left(1-R^{2}\right)V_{l}}{\frac{1}{n}tr\left(XX^{T}\right)}.$$

**Figure B1:**
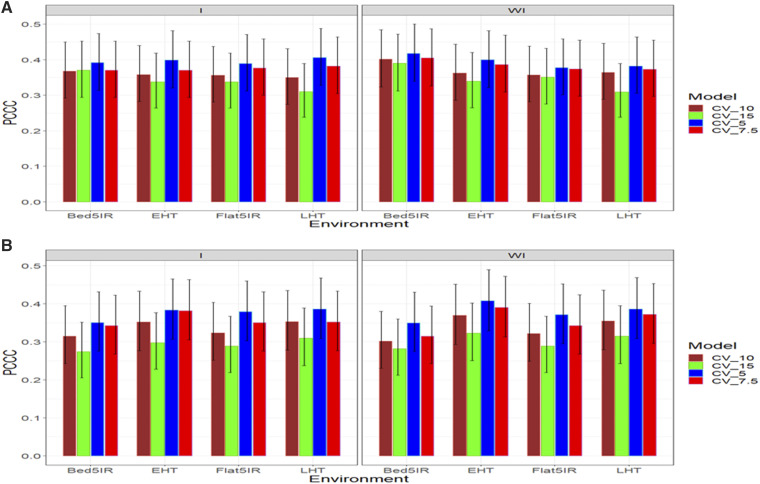
Prediction performance of the proposed MAPT model in terms of the proportion of cases correctly classified (PCCC) for traits: A) DTHD and B) DTMT of data set 1, under four values of coefficient of variation (CV). The smaller the CV, the more informative the prior. The left panel is with interaction (I) and the right panel is without interaction (WI).

**Figure B2:**
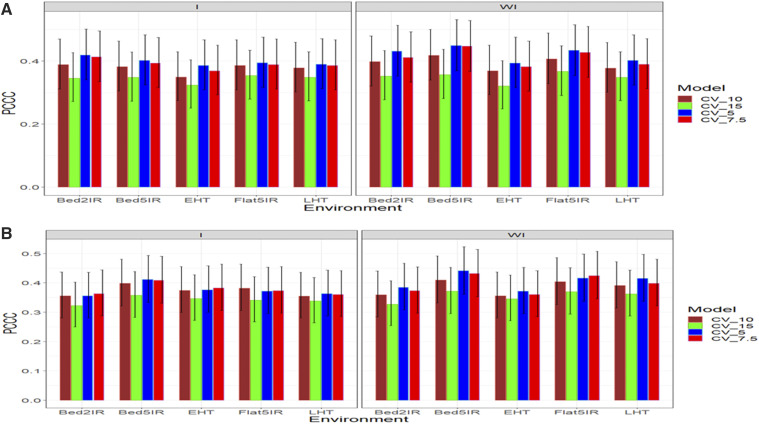
Prediction performance of the proposed MAPT model in terms of the proportion of cases correctly classified (PCCC) for traits: A) DTHD and B) DTMT of data set 2, under four values of coefficient of variation (CV). The smaller the CV, the more informative the prior. The left panel is with interaction (I) and the right panel is without interaction (WI).

**Figure B3:**
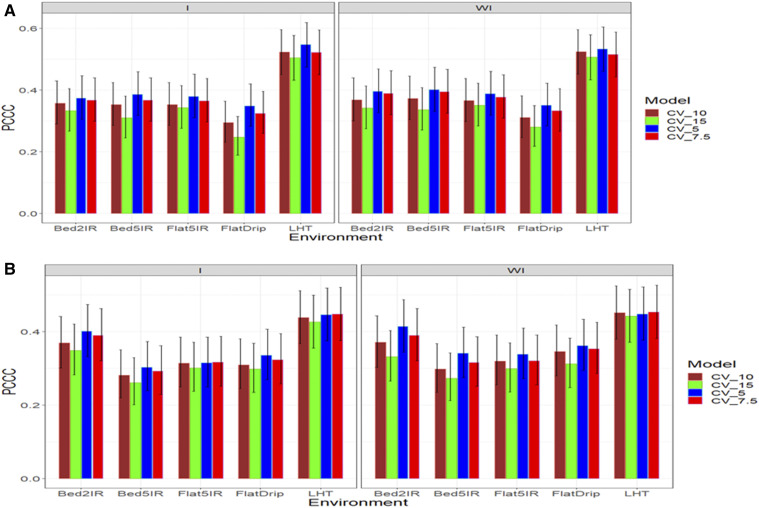
Prediction performance of the proposed MAPT model in terms of proportion of cases correctly classified (PCCC) for traits: A) DTHD and B) DTMT of data set 3, under four values of coefficient of variation (CV). The smaller the CV, the more informative the prior. The left panel is with interaction (I) and the right panel is without interaction (WI).

**Figure B4:**
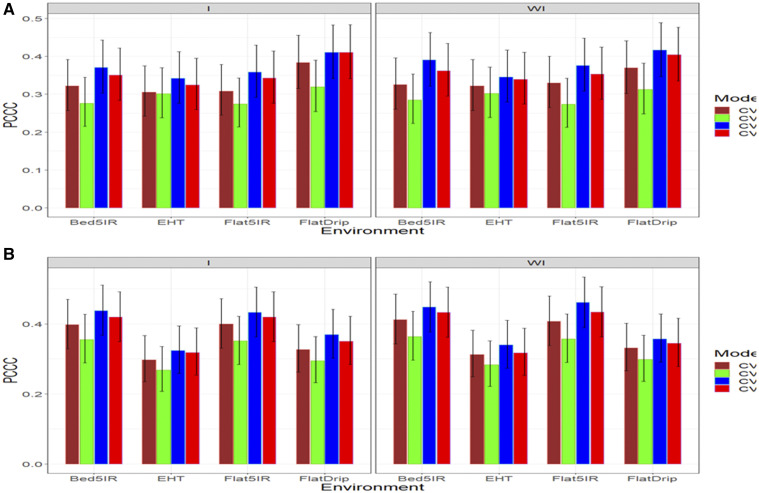
Prediction performance of the proposed MAPT model in terms of the proportion of cases correctly classified (PCCC) for traits: A) DTHD and B) DTMT of data set 4, under four values of coefficient of variation (CV). The smaller the CV, the more informative the prior. The left panel is with interaction (I) and the right panel is without interaction (WI).

But the concentration around this mean of the prior can be controlled for its coefficient of variation ($CV=\sqrt{\left[2⁄(v_{\beta }-4)]\right)}$ that only depends on the degrees of freedom $\left(v_{\beta }\right)$ parameter. For this reason, to evaluate the sensitivity of the prior distribution specification for $\sigma_{\beta }^{2}$, four values of CV were evaluated: 5%, 7.5%, 10% and 15% that resulted in the following degrees of freedom values ($v_{\beta }):$ 804, 369, 204 and 92.88, respectively. These different degrees of freedom (different values of CV) represent the amount of prior information. The larger (smaller) the CV (degrees of freedom=$v_{\beta })$, the less informative the prior distribution. Results are given in Figure B1 (Appendix B) for data set 1 for traits DTHD and DTMT. Figure B1 shows that the lower the CV (more informative prior), the better the prediction performance in both traits (DTHD and DTMT). However, a big difference in terms of prediction performance was not observed when the CV was increased from 5 to 7.5%; however, when the CV was increased to 15%, a drastic reduction in terms of prediction performance was observed. The performance of the proposed MAPT model with regard to the degree of informativeness of the prior for data set 2 (Figure B2), data set 3 (Figure B3) and data set 4 (Figure B4) was very similar and these plots (Figure B1 for data set 1) are displayed in Appendix B.

## Discussion

In this article, applications of the ECM algorithm for MAP estimation in the context of ordinal data for genomic-enabled prediction were introduced due to the need to implement GS with ever-increasing ordinal data sets. The general performance of the proposed Maximum *a posteriori* Threshold Genomic Prediction (MAPT) model was compared with that of the conventional Bayesian threshold genomic best linear unbiased prediction model, a multinomial Ridge regression model and the popular support vector machine. In terms of prediction performance, in most of the evaluated data sets, the proposed MAPT model was better than the conventional Bayesian threshold model and almost similar to the multinomial Ridge regression and support vector machine. However, in terms of implementation time, the MAPT model was almost always better than the multinomial Ridge regression, most of the time better than the conventional Bayesian Threshold Genomic prediction model and many times similar to or slightly better than the support vector machine model.

One advantage of the proposed MAPT model is that it is very stable and its implementation time in general is lower than that of models M1 and M3. The stability of the MAPT is attributed to the fact that it was built using the ECM algorithm that guarantees a monotonic increasing expected likelihood function, which means the iterative parameter estimation method will not have many divergence problems, as do conventional optimization methods. Another advantage of the proposed MAPT algorithm is that since it was built under the Bayes theorem framework, it allows incorporating prior information, but with the difference that instead of sampling from the distribution of the full conditionals, we only compute the posterior mode of the full conditional distributions.

Because we observed that the proposed MAPT using uninformative priors considerably underestimates the variance components compared to those obtained by the Bayesian Ordinal regression, to be able to successfully use the proposed method in the context of genomic prediction, we provided more informative priors for the variance component of the beta coefficients. The prior specification was done according to Pérez *et al.* (2010), who assume that a certain proportion of phenotypic variance is explained by the genotypic variance, but instead of equating the expected *a priori* genotypic variance divided by $\frac{1}{n}tr\left(XX^{T}\right)$ to the mode ($S_{\beta }/(v_{\beta }+2)$ of the prior distribution for the genetic variance, and solving for the scale parameter ($S_{\beta })$ for a fixed value of degrees of freedom ($v_{\beta })$, we equated the expected *a priori* genotypic variance divided by $\frac{1}{n}tr\left(XX^{T}\right)$ to the mean of the prior distribution for the genetic variance ($S_{\beta }/(v_{\beta }-2))$, and then we solved the scale parameter ($S_{\beta })$ for a fixed value of degrees of freedom ($v_{\beta })$. This approach allows control of the prior specification to be concentrated more around its mean prior phenotypic value with a desired coefficient of variation. It is important to point out that for a better performance of the proposed MAPT method, we always suggest scaling each independent variable.

As mentioned in the introduction, our method is different from the GEM method proposed by Kärkkäinen and Sillanpää (2013), which uses the conditional mean to update each parameter, while our proposed MAPT uses the conditional mode of each parameter, and the ECM algorithm implementation is based on a different latent variable than in the representation proposed by Ruud (1991).

On the other hand, an explanation of why many times the support vector machine was the best can be attributed to the fact that we implemented the support vector machine with a Gaussian kernel, while the other models (M1, M2 and M3) were implemented without a specific kernel (linear kernel). That is, the outstanding performance of the support vector machine can be attributed to the fact that the Gaussian kernel captures no linear patterns of the training data sets used that cannot be captured by models M1, M2 and M3, where linear kernels were used.

## Conclusions

In this research, we proposed an alternative method for the Bayesian threshold genomic best linear unbiased prediction model based on the MAP estimation method. The proposed method is simple, easy to implement and an efficient tool for learning parameters of a model since it was built using the Expected Conditional Maximization (ECM) for deriving the MAP for the conventional threshold genomic best linear unbiased prediction model. Our proposed maximum *a posteriori* threshold genomic prediction (MAPT) model was compared with the conventional Bayesian Threshold genomic best linear unbiased prediction model, the multinomial Ridge regression model and the support vector machine. We found that the proposed MAPT model was very competitive in terms of prediction performance with multinomial Ridge regression and the support vector machine which, in most data sets, outperformed the conventional Threshold genomic prediction model. However, in terms of implementation time, our proposed model (MAPT) and the support vector machine were the best, and the worst was the multinomial Ridge regression model, which although it produced a competitive prediction performance, its implementation time (computational resources) is extremely demanding. For these reasons, we encourage plant breeding scientists to benchmark the proposed method with other machine learning models for ordinal outcomes to get a better sense of the usefulness of our approach.

## Appendix A

The pdf of the gamma distribution with shape parameter s and rate parameter r:

$f_{S_{\beta }}\left(x;s,r\right)=\frac{r^{s}x^{s-1}}{\text{Γ}\left(s\right)}\exp\left(-rx\right)$. The mean, mode and the variance of this distribution are s/r, (s−1)/r and $s/r^{2}$, respectively. The probability density function (pdf) of the scaled inverse chi-square distribution with v degrees of freedom and scale parameter S, $\chi^{-2}\left(v,S\right)$, is given by

$$f\left(\sigma^{2};v,S\right)=\frac{{\left(\frac{S}{2}\right)}^{\frac{v}{2}}}{\text{Γ}\left(\frac{v}{2}\right){\left(\sigma^{2}\right)}^{1+\frac{v}{2}}}\exp(-\frac{S}{2\sigma^{2}}).$$

and the mean, mode, variance and coefficient of variation of this distribution are given by $\frac{S}{v-2}$, $\frac{S}{v+2}$, $\frac{2S^{2}}{{\left(v-2\right)}^{2}(v-4)}$ and $\sqrt{\frac{2}{v-4}}$ respectively. Note that if $\sigma^{2}∼\chi^{-2}\left(v,S\right)$ then $\sigma^{2}=\frac{1}{X}$ where $X∼G\left(v,\frac{S}{2}\right).$
